# Multi-dimensional characterization of prediabetes in the Project Baseline Health Study

**DOI:** 10.1186/s12933-022-01565-x

**Published:** 2022-07-18

**Authors:** Ranee Chatterjee, Lydia Coulter Kwee, Neha Pagidipati, Lynne H. Koweek, Priyatham S. Mettu, Francois Haddad, David J. Maron, Fatima Rodriguez, Jessica L. Mega, Adrian Hernandez, Kenneth Mahaffey, Latha Palaniappan, Svati H. Shah, the Project Baseline Health Study

**Affiliations:** 1 Department of Medicine, Duke University School of Medicine, 200 Morris Street, 3rd floor, NC 27701 Durham, USA; 2 Duke Molecular Physiology Institute, Durham, NC USA; 3 Department of Radiology, Duke University School of Medicine, Durham, NC USA; 4 Department of Ophthalmology, Duke University Medical Center, Durham, NC USA; 5 Department of Medicine, Stanford University, Palo Alto, CA USA; 6 Verily Life Sciences, San Francisco, CA USA; 7 Duke Clinical Research Institute, Duke University School of Medicine, NC Durham, USA

**Keywords:** Diabetes, Prediabetes, Risk factors

## Abstract

**Background:**

We examined multi-dimensional clinical and laboratory data in participants with normoglycemia, prediabetes, and diabetes to identify characteristics of prediabetes and predictors of progression from prediabetes to diabetes or reversion to no diabetes.

**Methods:**

The Project Baseline Health Study (PBHS) is a multi-site prospective cohort study of 2502 adults that conducted deep clinical phenotyping through imaging, laboratory tests, clinical assessments, medical history, personal devices, and surveys. Participants were classified by diabetes status (diabetes [DM], prediabetes [preDM], or no diabetes [noDM]) at each visit based on glucose, HbA1c, medications, and self-report. Principal component analysis (PCA) was performed to create factors that were compared across groups cross-sectionally using linear models. Logistic regression was used to identify factors associated with progression from preDM to DM and for reversion from preDM to noDM.

**Results:**

At enrollment, 1605 participants had noDM; 544 had preDM; and 352 had DM. Over 4 years of follow-up, 52 participants with preDM developed DM and 153 participants reverted to noDM. PCA identified 33 factors composed of clusters of clinical variables; these were tested along with eight individual variables identified *a priori* as being of interest. Six PCA factors and six *a priori* variables significantly differed between noDM and both preDM and DM after false discovery rate adjustment for multiple comparisons (q < 0.05). Of these, two factors (one comprising glucose measures and one of anthropometry and physical function) demonstrated monotonic/graded relationships across the groups, as did three *a priori* variables: ASCVD risk, coronary artery calcium, and triglycerides (q < 10^–21^ for all). Four factors were significantly different between preDM and noDM, but concordant or similar between DM and preDM: red blood cell indices (q = 8 × 10^-10^), lung function (q = 2 × 10^-6^), risks of chronic diseases (q = 7 × 10^-4^), and cardiac function (q = 0.001), along with *a priori* variables of diastolic function (q = 1 × 10^-10^), sleep efficiency (q = 9 × 10^-6^) and sleep time (q = 6 × 10^-5^). Two factors were associated with progression from prediabetes to DM: anthropometry and physical function (OR [95% CI]: 0.6 [0.5, 0.9], q = 0.04), and heart failure and c-reactive protein (OR [95% CI]: 1.4 [1.1, 1.7], q = 0.02). The anthropometry and physical function factor was also associated with reversion from prediabetes to noDM: (OR [95% CI]: 1.9 [1.4, 2.7], q = 0.02) along with a factor of white blood cell indices (OR [95% CI]: 0.6 [0.4, 0.8], q = 0.02), and the *a priori* variables ASCVD risk score (OR [95% CI]: 0.7 [0.6, 0.9] for each 0.1 increase in ASCVD score, q = 0.02) and triglycerides (OR [95% CI]: 0.9 [0.8, 1.0] for each 25 mg/dl increase, q = 0.05).

**Conclusions:**

PBHS participants with preDM demonstrated pathophysiologic changes in cardiac, pulmonary, and hematology measures and declines in physical function and sleep measures that precede DM; some changes predicted an increased risk of progression to DM. A factor with measures of anthropometry and physical function was the most important factor associated with progression to DM and reversion to noDM. Future studies may determine whether these changes elucidate pathways of progression to DM and related complications and whether they can be used to identify individuals at higher risk of progression to DM for targeted preventive interventions.

*Trial registration* ClinicalTrials.gov NCT03154346

**Supplementary Information:**

The online version contains supplementary material available at 10.1186/s12933-022-01565-x.

## Background

Prediabetes affects over one-third of the United States (U.S.) population and is associated with an increased risk of diabetes and cardiovascular disease (CVD) and higher health care utilization and costs [1–3]. However, prediabetes encompasses a wide range of abnormalities in glycemia, including impaired fasting glucose, impaired glucose tolerance, and impaired/elevated hemoglobin A1c (HbA1c), as well as a wide range of clinical, chemical, molecular, and pathophysiological abnormalities that are associated with varied risks of progression to more severe and clinically identifiable diabetes and CVD. Identification of the abnormalities associated with the different states of glycemia (normal glucose control, prediabetes, and diabetes) in the general U.S. population may help identify causal pathways of development and progression to more severe disease states. Additionally, identification of abnormalities associated with ‘high-risk’ prediabetes, or characteristics associated with a higher risk of progression to diabetes/CVD and complications, based on clinical, chemical, molecular, and pathophysiological data would allow for more targeted and effective preventive interventions.

The Project Baseline Health Study (PBHS) (ClinicalTrials.gov NCT03154346) is a unique, multicenter, prospective cohort harnessing advanced technological and digital capabilities for recruitment and data collection [4, 5]. The PBHS study performed deep phenotyping at in-person study visits, including medical history, physical function measures, imaging and biospecimen collection, as well as longitudinal digital health data, survey data, and annual follow-up. While prediabetes has been characterized through traditional cohort studies, in this study, we characterized participants with prediabetes compared with participants who had normal glycemic control or diabetes using the diverse and novel measures collected in the PBHS study. In addition, we identified biomarkers associated with progression from prediabetes to diabetes as well as reversion from prediabetes to normal glycemic control.

## Methods

### PBHS overview

The PBHS study is a multi-center, longitudinal cohort study designed to collect clinical, molecular, imaging, sensor, self-reported, psychological, and other health-related measurements to advance understanding of human health (ClinicalTrials.gov Identifier: NCT03154346). Description of the study design and procedures have been previously published [4]. Participants were recruited between 2017 and 2018, underwent a baseline visit at study enrollment, and were followed with in-person visits annually for 4 years. Participant selection was partially based on overall recruitment goals to include prespecified percentages of high-risk conditions in the cohort: 20% selected due to an elevated risk for primary CVD based on Framingham Risk Score and 2013 American College of Cardiology/American Heart Association (ACC/AHA) atherosclerotic cardiovascular disease (ASCVD) risk estimation equation; 20% selected due to an elevated risk for first-ever lung cancer based in part on cigarette smoking history; and 20% selected due to an elevated risk for first-ever breast/ovarian cancers based in part on being a carrier of a genetic mutation associated with breast/ovarian cancers; and all of these high-risk groups were selected relative to an age- and sex-matched distribution of risks observed in the U.S. national population [4]. The Institutional Review Board (IRB) at each clinical site approved the protocol, and all participants provided written informed consent.

### Data collection

At the initial in-person visit, participants underwent a broad array of testing and biospecimen collections, some of which were repeated at annual follow-up visits [5]. For this analysis, we used data from a range of PBHS assessments: self-reported demographics, medical history, and medications; clinical assessments, including vital signs and anthropometric measurements; physical performance testing; lung assessments, including pulmonary function tests and chest radiograph; cardiovascular assessments, including electrocardiograms; ankle-brachial index measurements, coronary calcium scan, echocardiogram, and stress echocardiogram; eye assessments, including optical coherence tomography and retinal photography; laboratory values measured from blood and urine biospecimens; psychological screening assessments collected either in-person or online; and step and sleep data collected via a Verily Study Watch. Laboratory data were not collected in a fasting state. This analysis was completed using data collected as of April 2021.

### Diabetes categories for PBHS participants

At each study visit, PBHS participants were categorized as: (1) no diabetes (noDM), defined as no self-reported history of prediabetes or diabetes, not on any diabetes medication, HbA1c < 5.7% (< 39 mmol/mol), and random glucose < 200 mg/dL; (2) prediabetes (preDM), defined as a self-reported history of prediabetes or a HbA1c of 5.7-6.4% (39–46 mmol/mol), not on any diabetes medication, and random glucose < 200 mg/dL; and (3) diabetes (DM), defined as a self-reported history of diabetes, HbA1c ≥ 6.5% (≥ 48 mmol/mol), random glucose ≥ 200 mg/dL, or on any diabetes medication. Participants who were defined as having prediabetes at the initial visit were defined as progressing to DM if they met the criteria for diabetes at any subsequent visit. Participants who were defined as having prediabetes at the initial visit were defined as reverting to noDM if they transitioned to, and remained in, a noDM status at any subsequent study visit.

### Statistical methods

Principal component analysis (PCA) was used to reduce the large number of correlated clinical variables into independent phenocluster factors composed of correlated variables. Overall, 122 clinical variables were considered for inclusion in PCA; variables with > 25% missing data were removed (N = 13), and remaining missing data were median-imputed. After performing PCA with varimax rotation on the resulting imputed data, we retained all factors with an eigenvalue > 1 (Kaiser criterion). Although all variables contributed to PCA factors, we considered variables with absolute loadings > 0.4 to be most important for a given factor, and we used these to describe each factor.

In addition to the derived PCA factors, we also analyzed eight individual variables of interest selected *a priori* given their high likelihood of association with diabetes (referred to as *“a priori”* variables hereafter): coronary artery calcium score (CAC) (≤ 100 vs. >100), ASCVD risk score, steps/day, hours of sleep/day, sleep efficiency, high-density lipoprotein cholesterol (HDL-C), triglycerides, and left ventricular diastolic function.

Univariate linear models were used to determine the association of each factor (and the eight *a priori* variables) with the three diabetes categories. Significant results adjusted for multiple comparisons (false discovery rate, FDR < 5%) from these initial models were then subjected to *post-hoc* tests of pairwise comparisons between all diabetes categories, with Tukey adjustment for multiple comparisons. To focus on clinically relevant relationships, factors that were not significantly different between DM and noDM were then removed, and each remaining significant factor (or *a priori* defined individual variable) was then placed in one of three possible groups based on its statistical relationship between groups: monotonic, concordant, or miscellaneous. Monotonic factors/variables showed a graded relationship among the three different diabetes categories, with significant differences between all pairwise comparisons (DM-preDM, DM-noDM, and preDM-noDM), with mean values for participants with prediabetes intermediate to those of the other two groups, suggesting a prediabetic state that is a precursor to diabetes. Concordant factors/variables were those that did not show significant differences between DM-preDM, but did show differences between preDM-noDM; this pattern reflects a situation in which prediabetes is similar to diabetes and suggests pathophysiologic changes that occur in the prediabetic state and remain similar in those individuals who progress to diabetes. Remaining miscellaneous factors showed a mix of patterns: significant differences between DM-preDM, but not between preDM-noDM (discordant), suggesting pathophysiologic changes that do not occur until progression to diabetes; preDM participants with mean values intermediate to, but not significantly different from, both DM and noDM; and preDM participants with mean values more extreme than, and significantly different from, both DM and noDM.

As the monotonic and concordant groups were of primary interest in this analysis, we sought to further characterize the variables driving the significant associations in these two groups using multivariable models adjusted for clinically relevant covariates. We identified the individual variables that were heavily loaded on monotonic and concordant factors and tested these, along with the monotonic and concordant *a priori* variables, in linear models adjusted for age, sex, race, body mass index (BMI), systolic blood pressure, and history of hypertension. For the variables that showed a significant association with the overall diabetes categories (p < 0.05), we examined differences between diabetes groups using pairwise comparisons.

We followed a similar approach to examine associations of PCA factors and *a priori* variables with progression from preDM to DM or for reversion from preDM to noDM. First, all PCA factors and *a priori* variables were tested for association with progression/non-progression or reversion/non-reversion status using univariate logistic regression models (FDR < 5%). Significant *a priori* variables and heavily loaded individual variables from significant factors were then tested in a multivariable model using the same adjustment variables as previously described.

## Results

### Study population and diabetes categories

A total of 2502 participants were recruited from four centers for the initial cohort of the PBHS study, 2501 of whom had sufficient data to define their glycemic status at study start, including 1605 with noDM, 544 with preDM, and 352 with DM. Selected enrollment characteristics of these PBHS participants are described in Table 1. The median ± intraquartile range (IQR) age of all PBHS participants was 50 ± 29 years, with a similar median age of 57 ± 22 years for those with preDM and DM compared with 46 ± 28 years of age for those with noDM. Approximately 55% of PBHS participants were women, and 16% self-reported Black/African American race, 10% Asian race, and 12% Hispanic ethnicity.

**Table 1:** Baseline characteristics of the Project Baseline Health Study (PBHS) cohort

	Overall	Diabetes	Prediabetes	No diabetes
n	2502	352	544	1605
*Age at enrollment (years), median [IQR] *	49.9 [35.0, 63.9]	58.2 [48.5, 68.7]	58.9 [46.9, 70.1]	43.2 [31.1, 59.1]
*Age category*				
18–44	1031 (41.2)	67 (19.0)	121 (22.2)	842 (52.5)
45–64	889 (35.5)	170 (48.3)	239 (43.9)	480 (29.9)
>=65	582 (23.3)	115 (32.7)	184 (33.8)	283 (17.6)
Female	1375 (55.0)	189 (53.7)	289 (53.1)	897 (55.9)
*Self-reported race*				
American Indian or Alaska Native	31 (1.2)	2 (0.6)	6 (1.1)	23 (1.4)
Asian	260 (10.4)	28 (8.0)	67 (12.3)	164 (10.2)
Black or African American	400 (16.0)	102 (29.0)	114 (21.0)	184 (11.5)
Native Hawaiian or Other Pacific Islander	27 (1.1)	5 (1.4)	5 (0.9)	17 (1.1)
Other	202 (8.1)	24 (6.8)	24 (4.4)	154 (9.6)
White	1582 (63.2)	191 (54.3)	328 (60.3)	1063 (66.2)
*Enrollment site*				
California Health and Longevity Institute	500 (20.0)	25 (7.1)	79 (14.5)	396 (24.7)
Durham	485 (19.4)	98 (27.8)	120 (22.1)	266 (16.6)
Kannapolis	515 (20.6)	118 (33.5)	122 (22.4)	275 (17.1)
Stanford	1002 (40.0)	111 (31.5)	223 (41.0)	668 (41.6)
Body mass index (kg/m^2^)	28.4 (6.9)	33.9 (8.2)	29.3 (6.7)	26.9 (5.9)
Waist circumference (cm)	92.7 (17.1)	107.1 (18.3)	95.9 (15.5)	88.5 (15.4)
Hemoglobin A1c (%)	5.7 (1.0)	7.3 (1.7)	5.9 (0.2)	5.2 (0.3)
Blood glucose (mg/dl)	97.7 (36.2)	148.7 (72.8)	94.1 (14.1)	87.6 (11.4)
Systolic blood pressure (mmHg)	123.2 (16.0)	129.5 (16.1)	126.7 (16.0)	120.7 (15.4)
History of hypertension	671 (26.8)	205 (58.2)	194 (35.7)	272 (16.9)
Total cholesterol (mg/dL)	184.5 (39.5)	173.1 (44.6)	189.7 (38.9)	185.2 (38.1)
LDL cholesterol (mg/dL)	99.6 (33.7)	87.4 (36.4)	103.6 (34.6)	100.7 (32.2)
HDL cholesterol (mg/dL)	58.7 (19.0)	49.9 (17.1)	59.3 (18.0)	60.4 (19.2)
Triglycerides (mg/dL)	135.1 (101.7)	187.1 (143.8)	138.3 (103.6)	122.3 (84.5)

Except where indicated, continuous variables are presented as the mean (SD), and categorical variables are presented as n (%). HDL, high-density lipoprotein; IQR, interquartile range; LDL, low-density lipoprotein

### Principal component analysis (PCA) factors associated with diabetes categories

After removing variables with > 25% missing data (N = 13), 109 variables were retained for inclusion in PCA analyses (Additional file 1: Table S1). For these analyses, an average of 4.9% of data were missing and imputed using the median for each variable. PCA identified 33 factors with eigenvalue > 1, each composed of clinically meaningful and interrelated loaded variables, which are described in Table 2 (factors were numbered according to their eigenvalues; larger eigenvalues represent a greater amount of the total variance that is explained by the factor). For example, factor 9 included variables related to glucose measures, diabetes, and inclusion in the high-risk CVD cohort. Factor 7 was composed of eye measurements, and factor 6 was composed of variables related to white blood cell (WBC) counts. Of these 33 factors, 24 differed significantly by diabetes status after adjustment for multiple comparisons; 12 of the 24 factors did not differ significantly between noDM and DM and were not considered further (Additional file 1: Table S2). The remaining 12 factors were categorized as monotonic, concordant, or miscellaneous (Table 3).

**Table 2:** Composition of principal component analysis (PCA) factors

Factor 1	Sit/rise score (0.60), one-leg balance (0.53), 6-minute walk (0.44), body mass index (BMI) (-0.68), waist circumference (− 0.71)
Factor 2	Forced vital capacity (FVC) (0.81), diffusing capacity for carbon monoxide (DLCO) (0.79), forced expiratory volume (FEV1) (0.78), hand grip strength (0.75), hemoglobin (0.67), hematocrit (0.65), red blood cells (abs) (0.61), 6-min walk (0.59), sex (− 0.77)
Factor 3	Smoking (0.74), selection for high-risk lung cancer cohort (0.61), selection for high-risk cardiovascular disease (CVD) cohort (0.44), vitamin D (− 0.42)
Factor 4	Neutrophils (%) (0.96), neutrophil/lymphocyte ratio (0.87), neutrophils (absolute count) (0.68), lymphocytes (absolute count) (− 0.48), lymphocytes (%) (− 0.96)
Factor 5	Calcium (0.80), albumin (0.76), serum protein (0.68)
Factor 6	White blood cells (WBC) (0.88), lymphocytes (0.79), neutrophils (0.66), monocytes (0.45)
Factor 7	Average retinal nerve fiber layer (RNFL) thickness (0.95), specific RNFL thicknesses: inferior quadrant (0.86), superior quadrant (0.82), nasal quadrant (0.70), total macular volume (0.59)
Factor 8	Mean corpuscular hemoglobin (MCH) (0.97), mean corpuscular volume (MCV) (0.89), mean corpuscular hemoglobin concentration (MCHC) (0.62), red blood cells (RBC) (− 0.47)
Factor 9	HbA1c (0.84), diabetes (0.80), glucose (0.77), selection for high-risk CVD cohort (0.47)
Factor 10	Left ventricular ejection fraction (LVEF) (0.93), left ventricular inner dimension end systole (LVIDs) (− 0.53)
Factor 11	Urine creatinine (0.88), specific gravity (0.87), urine albumin (0.81), pH (− 0.49)
Factor 12	Total cholesterol (0.94), LDL cholesterol (0.89)
Factor 13	Ratio of FEV1 and FVC (0.66), oxygen saturation (0.41), chronic obstructive pulmonary disease (COPD) (− 0.59)
Factor 14	Sodium (0.83), chloride (0.79)
Factor 15	Coronary artery disease (CAD) (0.79), history of myocardial infarction (MI) (0.78), coronary artery calcium (0.62)
Factor 16	Eosinophils (%) (0.95), eosinophils (abs) (0.95)
Factor 17	Serum creatinine (0.81), chronic kidney disease (CKD) (0.57), estimated glomerular filtration rate (eGFR) (− 0.69)
Factor 18	Aspartate transaminase (AST) (0.92), alanine transaminase (ALT) (0.88)
Factor 19	General anxiety disorder-7 (GAD-7) (0.88), patient health questionnaire-9 (PHQ-9) (0.86)
Factor 20	Basophils (%) (0.86), basophils (abs) (0.86)
Factor 21	Center subfield thickness (eye imaging) (0.77), vitreoretinal interface abnormalities (0.67), total macular volume (0.45)
Factor 22	Reticulocytes (%) (0.93), reticulocytes (abs) (0.92)
Factor 23	Platelets (0.66), mean platelet volume (− 0.85)
Factor 24	Alcohol use identification test-C (AUDIT-C) score (0.51)
Factor 25	Left ventricular mass index (LVMI) (0.65), left ventricular inner dimension end diastole (LVIDd) (0.60), left ventricular inner dimension end systole (LVIDs) (0.48), pulse (− 0.53)
Factor 26	Left ventricular cardiac index (0.90), left ventricular cardiac output (0.83), pulse (0.40)
Factor 27	Monocytes (%) (0.82), monocytes (abs) (0.75)
Factor 28	Thyroid-stimulating hormone (TSH) (0.60)
Factor 29	Systolic blood pressure (0.85), diastolic blood pressure (0.84)
Factor 30	Potassium (0.73)
Factor 31	Atrial fibrillation (0.44), selection for high-risk breast/ovarian cancer cohort (− 0.53)
Factor 32	Heart failure (0.71), C-reactive protein (CRP) (0.49)
Factor 33	Stroke (0.75)

All PCA factors with eigenvalue > 1 are shown, along with the component variables with absolute loadings > 0.4. Variables designated “(−)” are negatively loaded; all others are positively loaded. Factor loads for each variable presented in ( ). Abs: absolute count

**Table 3:** PCA factors significantly associated with diabetes groups in order of overall significance

Factor	Primary variables	Overall association with diabetes groups		Post-hoc pairwise comparisons, p†			
		p	q‡	DM vs. preDM	DM vs. noDM	PreDM vs. noDM	Category*
Factor 9	HbA1c, diabetes, glucose, selection for high-risk cardiovascular disease (CVD) cohort	< 10^− 70^	< 10^− 70^	< 10^− 70^	< 10^− 70^	< 10^− 70^	Monotonic
Factor 1	Sit/rise score, one-leg balance, 6-minute walk, body mass index (BMI) (−), waist circumference (−)	2 × 10^− 22^	1 × 10^− 21^	0.02	< 10^− 70^	< 10^− 70^	Monotonic
Factor 12	Total cholesterol, low-density lipoprotein (LDL) cholesterol	4 × 10^− 11^	2 × 10^− 10^	< 10^− 70^	1 × 10^− 5^	1 × 10^− 4^	Misc.
Factor 8	Mean corpuscular hemoglobin (MCH), mean corpuscular volume (MCV), mean corpuscular hemoglobin concentration (MCHC), red blood cells (RBC) (−)	2 × 10^− 10^	8 × 10^− 10^	0.4	6 × 10^− 4^	1 × 10^− 9^	Concordant
Factor 2	Forced vital capacity (FVC), diffusing capacity for carbon monoxide (DLCO), forced expiratory volume (FEV1), hand grip strength, hemoglobin, hematocrit, red blood cells (abs), 6-minute walk, sex	6 × 10^− 7^	2 × 10^− 6^	1	4 × 10^− 4^	2 × 10^− 5^	Concordant
Factor 32	Heart failure, C-reactive protein (CRP)	3 × 10^− 4^	6 × 10^− 4^	0.001	3 × 10^− 4^	1	Misc.
Factor 3	Smoking, selection for high-risk lung cancer cohort, selection for high-risk CVD cohort, vitamin D (−)	3 × 10^− 4^	7 × 10^− 4^	0.3	7 × 10^− 4^	0.05	Concordant
Factor 10	Left ventricular ejection fraction (LVEF), left ventricular inner dimension end systole (LVIDs) (−)	7 × 10^− 4^	0.001	0.9	0.05	0.002	Concordant
Factor 20	Basophils	0.002	0.004	0.002	0.02	0.2	Misc.
Factor 6	White blood cells (WBC), lymphocytes, neutrophils, monocytes	0.004	0.006	0.5	0.008	0.1	Misc.
Factor 11	Urine creatinine, specific gravity, urine albumin, pH (−)	0.01	0.02	0.1	0.008	0.7	Misc.
Factor 31	Atrial fibrillation, selection for high-risk breast/ovarian cancer cohort (−)	0.03	0.04	0.4	0.03	0.4	Misc.

DM, diabetes; preDM, prediabetes; noDM, no diabetes

Primary variables have absolute loadings >0.4 for the factor. Variables designated “(-)” are negatively loaded; all others are positively loaded

*Monotonic factors/*a priori* variables showed statistically significant differences between all pairwise comparisons (noDM-preDM, noDM-DM, and preDM-DM), with mean values for participants with prediabetes intermediate to those of the other two groups. Concordant factors/*a priori* variables did not show statistically significant differences between preDM-DM, but did show differences between preDM-noDM. Miscellaneous factors showed a mix of patterns: statistically significant differences between preDM-DM but not between preDM-noDM (discordant); preDM participants with mean values intermediate to, but not significantly different from, both DM and noDM; and preDM participants with mean values more extreme than, and significantly different from, both DM and noDM noDM.

†Adjusted for multiple comparisons using Tukey’s post-hoc test

‡False discovery rate (FDR)- adjusted p-values

**Table 4:** *A priori* defined individual variables associated with diabetes groups in order of overall significance

Variable	Overall association with diabetes group		Post-hoc pairwise comparisons, p†			
	p	q‡	DM vs. preDM	DM vs. noDM	PreDM vs. noDM	Category*
ASCVD risk score	1 × 10^− 70^	3 × 10^− 69^	2 × 10^− 11^	2 × 10^− 11^	2 × 10^− 11^	Monotonic
Coronary artery calcium > 100	3 × 10^− 26^	4 × 10^− 25^	7 × 10^− 5^	< 10^− 70^	2 × 10^− 9^	Monotonic
Triglycerides (mg/dl)	6 × 10^− 26^	6 × 10^− 25^	< 10^− 70^	< 10^− 70^	0.004	Monotonic
High density lipoprotein (mg/dl)	5 × 10^− 20^	3 × 10^− 19^	< 10^− 70^	< 10^− 70^	0.4	Misc.
Average daily number of steps	2 × 10^− 14^	9 × 10^− 14^	6 × 10^− 9^	4 × 10^− 11^	0.4	Misc.
Diastolic function score	2 × 10^− 11^	1 × 10^− 10^	0.2	2 × 10^− 8^	2 × 10^− 6^	Concordant
Average sleep efficiency (proportion of time asleep while in bed)	3 × 10^− 6^	9 × 10^− 6^	0.9	0.002	3 × 10^− 5^	Concordant
Average total sleep time (hr)	2 × 10^− 5^	6 × 10^− 5^	0.6	2 × 10^− 4^	0.004	Concordant

ASCVD, atherosclerotic cardiovascular disease; DM, diabetes; preDM, prediabetes; noDM, no diabetes

Primary variables have absolute loadings > 0.4 for the factor. Variables designated “(−)” are negatively loaded; all others are positively loaded

*Monotonic factors/*a priori* variables showed statistically significant differences between all pairwise comparisons (noDM-preDM, noDM-DM, and preDM-DM), with mean values for participants with prediabetes intermediate to those of the other two groups. Concordant factors/*a priori* variables did not show statistically significant differences between preDM-DM, but did show differences between preDM-noDM. Miscellaneous factors showed a mix of patterns: statistically significant differences between preDM-DM but not between preDM-noDM (discordant); preDM participants with mean values intermediate to, but not significantly different from, both DM and noDM; and preDM participants with mean values more extreme than, and significantly different from, both DM and noDM

†Adjusted for multiple comparisons using Tukey’s post-hoc test

‡False discovery rate (FDR)-adjusted p-values

Two factors differed significantly between all three pairwise diabetes groups with a monotonic relationship across categories: factor 9, composed of heavily loaded variables of HbA1c, diabetes, glucose, inclusion in the high-risk CVD cohort (q [FDR-adjusted p] < 10^− 21^), and factor 1, composed of sit/rise score, one-leg balance, 6-min walk, BMI (−), and waist circumference (−) (q = 1.3 × 10^− 21^). A “(−)” next to a given variable indicates a negative load on the relevant factor.

Four factors were concordant between DM and preDM (i.e. similar between preDM and DM but both different from noDM): factor 8, composed of red blood cell (RBC) morphology (q = 8.3 × 10^− 10^); factor 2, composed of lung function measures, RBC measures, and physical function measures (q = 2.0 × 10^− 6^); factor 3, composed of smoking, high-risk lung cancer cohort, high-risk CVD cohort, and vitamin D (−) (q = 7.0 × 10^− 4^); and factor 10, composed of echocardiographic measures of cardiac structure, including left ventricular ejection fraction (LVEF) and left ventricular inner dimension end systole (LVIDs) (−) (q = 0.001) (Table 3).

Of the factors that were in the miscellaneous category and not analyzed further, two factors were discordant between DM and preDM (i.e. similar between preDM and noDM but both different from DM): factor 32, composed of a history of heart failure and high-sensitivity C-reactive protein (hsCRP) (q = 6.1 × 10^− 4^), and factor 20, composed of basophils (q = 0.004). Three factors differed significantly only between DM and noDM, including factor 6 (measures of WBC), factor 11 (urinary measures), and factor 31 [atrial fibrillation and selection for a high risk of ovarian cancer (−)]. One factor, factor 12, which included variables of cholesterol and low-density lipoprotein cholesterol, differed significantly between all three comparisons, but with higher levels in the preDM group than both the noDM and DM groups (Table 3).

### Association of a priori defined individual variables of interest with diabetes categories

Of the variables identified *a priori* to be of interest based on their known strong relationship with prediabetes and diabetes, all eight differed significantly between diabetes categories overall (Table 4). In assessments of two-way comparisons, three of these variables demonstrated a monotonic relationship across diabetes categories: ASCVD score (q = 2.8 × 10^− 69^), CAC score (q = 3.7 × 10^− 25^), and triglycerides (q = 5.9 × 10^− 25^). Three variables were concordant between DM and preDM: mean hours of sleep/night (q = 6.1 × 10^− 5^), mean sleep efficiency (q = 8.8 × 10^− 6^), and diastolic function (q = 1.2 × 10^− 10^) (Table 4). The other variables of interest, HDL-C and mean steps/day, were discordant between DM and pre-DM (i.e. pre-DM was similar to noDM).

### Multivariable models for individual variables within PCA factors Associated with Diabetes

To assess whether these relationships were independently associated with diabetes groups after adjustment for relevant covariables, we deconstructed each PCA factor that demonstrated a monotonic or concordant relationship across diabetes categories into the variables most heavily loaded on each factor (absolute factor load > 0.4). The majority of these individual variables (21 out of 25) were significantly different across diabetes groups in multivariable models adjusted for age, sex, race, body mass index, systolic blood pressure, and history of hypertension (Additional file 1: Table S3a). Of these 21 variables, four demonstrated a monotonic relationship in multivariable models: blood glucose, HbA1c, BMI, and forced vital capacity (FVC). Eight of these variables were concordant between DM and preDM, including five variables related to RBC measures and three variables related to lung function or smoking status (forced expiratory volume [FEV1], current smoker, and selection for the high-risk lung cancer cohort). Seven variables were discordant between DM and preDM, including measures of physical function, waist circumference, LVID, and selection for the high-risk CVD cohort.

Of the six *a priori* defined individual variables of interest that were significantly associated with diabetes categories, three remained significant in multivariable models: ASCVD risk score (p = 1.7 × 10^− 37^), which differed significantly between all three groups with the preDM group having a slightly lower score compared with the noDM group; and triglycerides (p = 4.0 × 10^− 10^) and CAC score (p = 0.001), both of which were discordant between preDM and DM (Additional file 1: Table S3b).

### PCA factors and variables associated with progression from prediabetes to diabetes

Of 544 individuals with preDM at study start, 52 (9.6%) developed diabetes over a median ± IQR of 1 ± 2 years and up to 4 years of follow-up. In analyses of the 33 PCA factors, two were significantly associated with progression from prediabetes to diabetes in univariate models after adjustment for multiple comparisons (Table 5): factor 1 (anthropometric and physical function measures) and factor 32 (history of heart failure and hsCRP) (q = 0.04 for each). Of the individual variables heavily loaded on these two factors, only BMI and waist circumference remained significantly associated with increased odds of progression from prediabetes to diabetes in multivariable models: BMI odds ratio (OR) (95% confidence interval, CI) 1.1 (1.05,1.16), p = 4.5 × 10^− 5^ and waist circumference OR (95% CI) 1.06 (1.02, 1.11), p = 0.007.

**Table 5:** Factors and individual *a priori* variables associated with progression from prediabetes to diabetes

PCA factor or individual variable tested	Primary variables in PCA factor	Univariate model			Adjusted model †	
		OR (95% CI)	p	>q*	OR (95% CI)	p
Factor 1	Sit/rise score, one-leg balance, 6-minute walk, body mass index (BMI) (−), waist circumference (−)	0.6 (0.5,0.9)	0.002	0.04	0.7 (0.5,0.9) **	0.01
Factor 32	Heart failure, C-reactive protein (CRP)	1.4 (1.1,1.7)	0.002	0.04	1.2 (0.9,1.5)	0.2

OR, odds ratio; CI, confidence interval

OR > 1: higher levels of the factor are associated with higher odds of progression from prediabetes to diabetes; OR < 1: higher levels of the factor are associated with lower odds of progression from prediabetes to diabetes

† Adjusted for age, sex, race, body mass index (BMI), systolic blood pressure, and history of hypertension

*False discovery rate (FDR)-adjusted p-values

** Factor 1 model is not adjusted for BMI, which is a heavily-loaded component of the factor

**Table 6:** Factors and individual *a priori* variables associated with reversion from prediabetes to no diabetes

PCA factor or individual variable tested	Primary variables in PCA factor	Univariate model			Adjusted model †	
		OR (95% CI)	**p**	q*	OR (95% CI)	**p**
Factor 1	Sit/rise score, one-leg balance, 6-minute walk, body mass index (BMI) (−), waist circumference (−)	1.4 (1.2,1.7)	0.0007	0.02	1.2 (1.0,1.5) **	0.04
Factor 6	White blood cells (WBC), lymphocytes, neutrophils, monocytes	0.7 (0.6,0.9)	0.0009	0.02	0.6 (0.5, 0.8)	0.0001
ASCVD risk score		0.7 (0.6,0.9)^@^	0.001	0.02	1.0 (0.7, 1.4)	1.0
Triglycerides (mg/dl)		0.9 (0.8,1.0)^#^	0.005	0.05	0.9 (0.9, 1.0)	0.03

OR, odds ratio; CI, confidence interval

OR > 1: higher levels of the factor/variable are associated with higher odds of progression from prediabetes to diabetes; OR < 1: higher levels of the factor/variable are associated with lower odds of progression from prediabetes to diabetes

† Adjusted for age, sex, race, body mass index (BMI), systolic blood pressure, and history of hypertension

*False discovery rate (FDR)-adjusted p-values

** Factor 1 model is not adjusted for BMI, which is a heavily-loaded component of the factor

^@^ OR for a 0.1 unit increase in ASCVD risk score

^#^ OR for a 25 mg/dl increase in triglycerides

### PCA factors and variables associated with reversion from prediabetes to no diabetes

Of 544 individuals with preDM at study start, 153 reverted to noDM (i.e. reverted to and stayed in a noDM state at some post-enrollment visit, and did not progress to diabetes at any timepoint). In analyses of the 33 PCA factors, two were significantly associated with reversion in univariate models after adjustment for multiple comparisons (Table 6): factor 1 (anthropometric and physical function measures, OR (95% CI) 1.4 (1.2, 1.7), q = 0.02); and factor 6 (white blood cells, OR (95% CI) 0.7 (0.6, 0.9), q = 0.02), along with two *a priori* variables: ASCVD risk score, OR (95% CI) 0.7 (0.6, 0.9) per 0.1 increase in score and triglycerides, OR (95% CI) 0.9 (0.84, 0.96). Individual variables heavily loaded on these two factors and *a priori* variables that remained significantly associated with reversion in multivariable models are presented in Additional file 1: Table S4.

## Discussion

Leveraging the deeply phenotyped PBHS cohort, we identified multiple clinical, imaging, laboratory, physical function, and digital health features associated with prediabetes and risk of progression from prediabetes to diabetes and odds of reversion to a normal glycemic state. Specifically, we found pathophysiologic changes in measures of anthropometry, physical function, sleep measures, hematologic indices, cardiac structure, and lung function that occur across the spectrum of diabetic states. Some of these changes are monotonic and develop across the continuum of diabetic states, some pathophysiologic changes develop in the prediabetic state and are concordant with the changes found with diabetes, and some changes do not occur until diabetes develops (i.e. are not present in the preDM state). While many of the pathophysiologic changes that we found were expected, such as changes in glucose measures and measures of adiposity, some of the changes in participants with prediabetes, including changes in RBC measures, have not been previously described. These findings support the use of deep phenotyping data to better understand prediabetes and the potential biological mechanisms of progression to diabetes/CVD in a systemic multi-organ system context.

Features demonstrating monotonic relationships across the spectrum of no diabetes through prediabetes to diabetes suggest that dysregulated clinical features are related to diabetes risk across a continuum. We found two PCA factors that demonstrated these monotonic relationships, including one factor composed of measures of glucose control; however, this was expected given that levels of glucose control were used to define the diabetes groups and given the known monotonic relationship of glycemia across stages of diabetes. The second factor included anthropometric measures of BMI and waist circumferences and measures of physical function, including scores of single-leg balance, age-adjusted 6-minute walk test, sit-rise score, and hand grip strength. While many studies have confirmed poorer physical function in people with diabetes [6], fewer studies have evaluated physical function in people with prediabetes. Both a cross-sectional British study and a longitudinal Finnish study have found that higher post-prandial glucose levels, even within normal ranges and impaired glucose tolerance ranges, are associated with lower physical function scores, including measures related to strength, endurance, and flexibility [7, 8]. Several potential causal mechanisms for these findings of lower physical performance include increased muscle protein degradation related to insulin resistance; increased inflammation associated with conditions of prediabetes and obesity, causing negative effects on skeletal muscle; increased fat infiltration of muscle; increased mitochondrial dysfunction or impaired blood flow due to hyperglycemia; and/or the presence of peripheral neuropathy [9–11]. In addition to these potential mechanisms of hyperglycemia or insulin resistance impacting muscular function, people with prediabetes may have reduced physical activity, as demonstrated by a reduced step count in the PBHS study. This reduced activity could lead to increased weakness and worse muscular function and may contribute to the development of prediabetes and progression to diabetes. Therefore, the directionality of the association between poorer physical function and prediabetes is not clear. In our longitudinal analysis, the finding that this same factor was associated with incident diabetes risk, with higher physical function scores and lower BMI and waist circumference associated with a lower risk of diabetes and higher odds of reversion to normal glucose control, supports the public health position that interventions to improve or maintain physical function in people with prediabetes may prevent transition from prediabetes to diabetes [12–14].

We also found clinical features that demonstrated a concordant relationship, with similar levels/prevalence in the prediabetes and diabetes groups but significantly different levels in the no diabetes group, suggesting that dysregulation of these variables occurs earlier in the diabetes spectrum and are already dysregulated in patients with prediabetes. These factors included variables associated with RBC morphology, measures of lung function, and measures of cardiac function/structure. RBC measures within the factor demonstrating this concordant relationship included mean corpuscular volume (MCV), mean corpuscular hemoglobin (MCH), and mean corpuscular hemoglobin concentration (MCHC), with values reflecting smaller RBC size being associated with prediabetic and diabetic states. These results are consistent with findings from prior experimental and clinical studies; our findings extend these smaller prior studies into a larger study and into prediabetes for the first time to our knowledge. Ex vivo studies have found that RBCs undergo changes when exposed to higher glucose concentrations, with evidence of hemoglobin oxidation and changes in the cell membrane consistent with loss of elasticity [15]. Small clinical studies comparing the RBCs of patients with diabetes with those of healthy controls have demonstrated differences in RBC morphology between the two groups [16]. The patients with diabetes demonstrated an increased number of RBCs with irregular shapes and smaller diameters, including an increased number of spherocytes with smaller MCV, which have been found to be higher in patients with type 1 and type 2 diabetes [16, 17]. RBCs of patients with diabetes have also been found to have more rigid cellular membranes, thought to be due to protein glycosylation. This increased stiffness or decreased deformability has been found to result in a shorter lifespan of RBCs, and hence, could explain the higher reticulocyte count that was observed in our participants with prediabetes and diabetes compared with participants with normal glucose control and that was found to be a predictor of progression to diabetes [16]. These changes in RBC structure, in particular the decreases in deformability, are thought to contribute to microvascular complications related to diabetes. The finding that these structural changes in RBCs may occur before the development of diabetes and are evident in the prediabetes stage is of interest and may contribute to the increased risk of chronic disease, including CVD, in patients with prediabetes, warranting further study.

Other factors demonstrating these concordant relationships included a factor with measures from pulmonary function testing. Analysis of directionality of loadings within this factor showed lower FVC and FEV1 in participants with prediabetes and diabetes compared with participants without diabetes. These results are consistent with prior studies that have shown differences in lung function measures between glycemic groups, with reduced lung function associated with higher HbA1c levels and diabetic status [18]. Longitudinal cohort studies have also identified greater declines in lung function in individuals with diabetes compared with individuals with normal glucose control, suggesting that declines in lung function could be due to diabetes. Other longitudinal studies have found declines in lung function to be predictors of diabetes risk, with stronger associations in certain populations [18–20]. The underlying mechanism of this increased risk includes central obesity, which is often present in conditions of prediabetes and diabetes and results in reduced lung function. Other hypotheses include intrauterine exposures that result in low birthweight, reduced lung function, and increased diabetes risk; the potential for high glucose levels to cause reduced lung function through glycosylation of lung tissue, thereby reducing lung elasticity and function; and high glucose levels that result in microvascular damage, inflammation, and neuropathy, affecting respiratory muscles [18].

A factor that included measures of left ventricular ejection fraction (LVEF) and left ventricular inner dimension systole (LVIDs) also had a concordant relationship in our cross-sectional analyses, with individuals with prediabetes and diabetes having progressively smaller LVIDs and progressively greater LVEF than individuals without diabetes. The individual variable of diastolic function score showed a similar relationship, with higher scores in participants with prediabetes and diabetes although this did not remain significant in multivariable models. However, in participants with prediabetes, smaller LVIDs and higher LVEF at baseline were associated with a lower risk of diabetes in the context of this factor in our longitudinal model. Our longitudinal results are consistent with prior studies. For example, meta-analyses have demonstrated a high prevalence of diastolic dysfunction in individuals with diabetes [21, 22]. Fewer studies have examined cardiac structural changes in people with prediabetes. An analysis of the Atherosclerosis Risk In the Community (ARIC) cohort did find progressive increases in left ventricular mass, worsening of diastolic function, and slight worsening of systolic function associated with increases in HbA1c values and diabetic/glycemic status [23]. Our results suggest that left ventricular remodeling occurs early in the diabetes continuum and further highlights the importance of interventions in individuals with prediabetes.

Importantly, given the longitudinal nature of the PBHS study, we were able to analyze predictors of early progression from prediabetes to diabetes and reversion from prediabetes to no diabetes. Specifically, as previously discussed, we found that the factor composed of anthropometric and physical function measures (factor 1) that demonstrated a monotonic relationship across diabetes groups was a predictor of progression to diabetes as well as a predictor of reversion to normal glucose control. As previously detailed, this finding is perhaps expected given prior studies; our results further support earlier interventions to promote physical function to prevent diabetes progression and to increase the likelihood of reversion to normal glucose control. We also found that a factor composed of a history of heart failure and hsCRP (factor 32) predicted progression to diabetes. Although this factor was similar between participants with prediabetes and diabetes at baseline, it predicted progression to diabetes, suggesting that inflammation could be an early marker of and contributor to increased diabetes risk, as has been found in prior studies [24].

We found that a factor of WBC measures was associated with reversion to normal glucose control, with higher WBC measures associated with a lower likelihood of reversion. Elevations in WBC counts occur in inflammatory states, and certain WBC types, particularly lymphocytes, can release inflammatory cytokines, such as interferons and interleukins, which are markers of and contributors to inflammation. Higher WBC counts have been found to be associated with type 2 diabetes, prediabetes, and metabolic syndrome [25–27]. While we did not find the factor of WBC to be a significant predictor of diabetes risk, the finding that higher WBC counts may impede reversion to normal glucose control suggests that WBCs contribute to glucose dysregulation. In a separate analysis comparing participants who reverted to normoglycemia with the subset of participants who progressed to diabetes, this WBC factor remained associated with reversion to normoglycemia, along with the physical function and anthropometry factor (data not shown). More detailed study of these associations through use of advanced technology, including flow cytometry, may determine more precise mechanisms through which WBCs and inflammatory cytokines lead to glucose dysregulation.

Our findings of associations between the *a priori* variables and diabetes status are also of interest. The monotonic associations of ASCVD risk score, CAC score, and triglycerides across the spectrum of diabetes groups are consistent with prior studies that have shown that people with prediabetes are at increased risk of CVD [1, 28]. Finally, the concordant associations between sleep sensor data and diabetes status are noteworthy. PBHS participants with prediabetes and diabetes had similarly lower sleep efficiency and fewer sleep hours compared with participants with no diabetes. Similar risk factors, particularly obesity, are shared between diabetes/prediabetes and certain sleep conditions, including obstructive sleep apnea (OSA), which are associated with lower sleep efficiency. Additionally, conditions of sleep disturbances, including short as well as overly long sleep times, insomnia, OSA, and abnormal sleeping schedules, have been found to be associated with a higher risk of diabetes, comparable to other more traditional diabetes risk factors [29]. The finding in our study that these measures of sleep, including sleep time and efficiency, which were measured with a personal sensor device, are similarly abnormal in participants with prediabetes and diabetes deserves further study to determine if these measures identify people at highest risk of diabetes and its complications.

Our results demonstrate one of the strengths of our study, that of the robust depth of data collection that was conducted in the PBHS cohort, particularly at the baseline visit and the close follow-up of participants over 4 years. Data were collected from novel data sources, including self-report through the study portal and wearable sensor data, which are becoming more commonly used. The limitations of our study include our categorization of diabetic/glycemic status, which we based on self-report of diabetes, use of any medication, including metformin, considered consistent with a diabetes diagnosis, and collection of HbA1c and random glucose measures. Fasting glucose and 2-hour oral glucose challenge tests were not conducted during the study and would have allowed for more accurate categorization of diabetes and the different types of prediabetes. Additionally, while a wide array of data were collected in PBHS, many other potential risk factors of diabetes and CVD and measures of CVD intermediate outcomes were not assessed. Other studies have found associations between characteristics of participants with prediabetes and increased risk of diabetes/CVD that could not be verified in this study [30–32]. Further verification of these findings is warranted in prospective and larger studies.

## Conclusions

In conclusion, participants with prediabetes in the PBHS cohort have many pathophysiologic findings that are also present in people with diabetes. Further study of the associations between these abnormalities and downstream complications may help better elucidate (1) biological pathways of progression to diabetes/CVD and its complications, (2) potential targets for intervention to prevent progression and complications, and (3) identification of patients with higher-risk prediabetic states that may be targeted for more intensive preventive interventions.

## Supplementary Information

**Additional file 1: Table S1.** All clinical variables included in the calculation of PCA factors. **Table S2.** PCA factors significantly associated overall with diabetes groups, with no differences between DM and noDM. **Table S3a.** Individual variables with high loadings in PCA factors associated with diabetes categories in the multivariable models. **Table S3b.** *A priori* individual variables associated with diabetes categories in the multivariable models. **Table S4.** Individual variables with high loadings in PCA factors associated with progression to diabetes and reversion to no diabetes.

## Data Availability

The Project Baseline Health Study data will be available to qualified researchers for exploratory analysis after the data are adequately curated and initial planned primary manuscripts are written. Qualified external researchers will be able to apply through applications reviewed by the Proposal Review and Publications Committee and Scientific Executive Committee.

## Abbreviations

ASCVD: Atherosclerotic cardiovascular disease
BMI: Body mass index
CAC: Coronary artery calcium
CVD: Cardiovascular disease
DLCO: Diffusing capacity for carbon monoxide
FDR: False discovery rate
FEV1: Forced expiratory volume
FVC: Forced vital capacity
HDL-C: High-density lipoprotein cholesterol
IRB: Institutional Review Board
LV: Left ventricular
LVEF: Left ventricular ejection fraction
LVID: Left ventricular inner dimension
MCV: Mean corpuscular volume
MCH: Mean corpuscular hemoglobin
MCHC: Mean corpuscular hemoglobin concentration
PBHS: Project Baseline Health Study
PCA: Principal component analysis
RBC: Red blood cell
SD: Standard deviation
WBC: White blood cell
