# Supplementary material for: Multi-dimensional characterization of prediabetes in the Project Baseline Health Study
Source: Cardiovasc Diabetol. 2022 Jul 18;21:134. doi: 10.1186/s12933-022-01565-x (PMC9295520; doi:10.1186/s12933-022-01565-x)
Supplement: Supplementary file 1 — Additional file 1: Table S1. All clinical variables included in the calculation of PCA factors. Table S2. PCA factors significantly associated overall with diabetes groups, with no differences between DM and noDM. Table S3a. Individual variables with high loadings in PCA factors associated with diabetes categories in the multivariable models. Table S3b. A priori individual variables associated with diabetes categories in the multivariable models. Table S4. Individual variables with high loadings in PCA factors associated with progression to diabetes and reversion to no diabetes. [file 12933_2022_1565_MOESM1_ESM.docx]

**ADDITIONAL FILES**

**Additional Table S1.** All clinical variables included in the calculation of PCA factors

|  | | Diabetes | | Prediabetes | | No diabetes |
| --- | --- | --- | --- | --- | --- | --- |
|  | | **N=352** | | **N=544** | | **N=1605** |
| *Self-report and medical history* | |  | |  | |  |
| Age at enrollment | | 57.1 (14.1) | | 57.3 (15.2) | | 46.0 (17.2) |
| Female | | 189 (53.7) | | 289 (53.1) | | 897 (55.9) |
| Self-reported race | |  | |  | |  |
| American Indian or Alaska Native | | 2 (0.6) | | 6 (1.1) | | 23 (1.4) |
| Asian | | 28 (8.0) | | 67 (12.3) | | 164 (10.2) |
| Black or African American | | 102 (29.0) | | 114 (21.0) | | 184 (11.5) |
| Native Hawaiian or Other Pacific Islander | | 5 (1.4) | | 5 (0.9) | | 17 (1.1) |
| Other | | 24 (6.8) | | 24 (4.4) | | 154 (9.6) |
| White | | 191 (54.3) | | 328 (60.3) | | 1063 (66.2) |
| Smoking status (current vs. former/never) | | 64 (18.2) | | 97 (17.8) | | 176 (11.0) |
| History of cancer | | 59 (16.8) | | 98 (18.0) | | 202 (12.6) |
| History of chronic kidney disease | | 8 (2.3) | | 7 (1.3) | | 7 (0.4) |
| History of hyperlipidemia | | 151 (42.9) | | 165 (30.3) | | 222 (13.8) |
| History of stroke | | 9 (2.6) | | 10 (1.8) | | 12 (0.7) |
| History of coronary artery disease | | 22 (6.2) | | 31 (5.7) | | 30 (1.9) |
| History of chronic obstructive pulmonary disease | | 28 (8.0) | | 22 (4.0) | | 25 (1.6) |
| History of heart failure | | 8 (2.3) | | 5 (0.9) | | 6 (0.4) |
| History of myocardial infarction | | 10 (2.8) | | 15 (2.8) | | 17 (1.1) |
| History of peripheral vascular disease | | 7 (2.0) | | 6 (1.1) | | 15 (0.9) |
| History of diabetes | | 276 (78.4) | | 0 (0.0) | | 0 (0.0) |
| History of hypertension | | 205 (58.2) | | 194 (35.7) | | 272 (16.9) |
|  |  | |  | |  |  |
| *Cardiac imaging* | |  | |  | |  |
| Best available left ventricular ejection fraction from biplane, single and visual read data from resting echocardiogram | | 59.5 (5.0) | | 59.1 (4.7) | | 58.5 (3.8) |
| 2D biplane left ventricular ejection fraction (%) | | 59.3 (5.0) | | 58.9 (4.6) | | 58.4 (3.8) |
| Left ventricular mass index | | 73.3 (16.8) | | 73.2 (19.2) | | 68.1 (16.2) |
| Left ventricular inner dimension end diastole (cm) | | 4.4 (0.5) | | 4.4 (0.5) | | 4.5 (0.5) |
| Left ventricular inner dimension end systole (cm) | | 2.7 (0.5) | | 2.7 (0.5) | | 2.8 (0.4) |
| Left ventricular cardiac output (l/min) | | 5.1 (1.4) | | 4.7 (1.3) | | 4.7 (1.2) |
| Left ventricular cardiac index | | 2.5 (0.6) | | 2.4 (0.5) | | 2.5 (0.5) |
| Diastolic function score | |  | |  | |  |
| 0 | | 196 (55.7) | | 358 (65.8) | | 1243 (77.4) |
| 1 | | 27 (7.7) | | 39 (7.2) | | 37 (2.3) |
| 2 | | 13 (3.7) | | 14 (2.6) | | 17 (1.1) |
| 3 | | 2 (0.6) | | 4 (0.7) | | 7 (0.4) |
| NA | | 114 (32.4) | | 129 (23.7) | | 301 (18.8) |
| Coronary artery calcium | | 242.9 (559.6) | | 192.9 (636.9) | | 73.6 (304.2) |
| Atrial fibrillation | | 3 (0.9) | | 4 (0.7) | | 6 (0.4) |
| *Clinical measures* | |  | |  | |  |
| Body mass index (kg/cm2) | | 33.9 (8.2) | | 29.3 (6.7) | | 26.9 (5.9) |
| Systolic blood pressure readings (mmHg) | | 129.5 (16.1) | | 126.7 (16.0) | | 120.7 (15.4) |
| Diastolic blood pressure (mmHg) | | 78.1 (9.6) | | 77.1 (10.1) | | 74.9 (9.8) |
| Ankle brachial index | | 1.1 (0.1) | | 1.1 (0.1) | | 1.1 (0.1) |
| Pulse | | 73.3 (12.4) | | 65.9 (11.1) | | 66.6 (11.2) |
| Oxygen saturation | | 97.9 (1.7) | | 98.4 (1.5) | | 98.7 (1.6) |
| Respiratory rate | | 16.2 (2.7) | | 15.7 (2.6) | | 15.5 (2.4) |
| Waist circumference (cm) | | 107.1 (18.3) | | 95.9 (15.5) | | 88.5 (15.4) |
| Atherosclerotic cardiovascular disease (ASCVD) risk score | | 0.2 (0.2) | | 0.1 (0.1) | | 0.0 (0.1) |
| *Eye imaging* | |  | |  | |  |
| Total macular volume | | 10.0 (0.6) | | 10.0 (0.5) | | 10.1 (0.5) |
| Center subfield thickness (center 1mm) | | 258.6 (33.8) | | 257.6 (26.2) | | 259.3 (24.4) |
| Average retinal nerve fiber layer thickness | | 89.8 (13.3) | | 91.0 (11.5) | | 93.5 (10.8) |
| Retinal nerve fiber layer superior quadrant thickness | | 110.5 (21.9) | | 112.3 (17.7) | | 116.6 (17.6) |
| Retinal nerve fiber layer nasal quadrant thickness | | 69.3 (12.2) | | 71.2 (13.2) | | 71.8 (12.6) |
| Retinal nerve fiber layer inferior quadrant thickness | | 116.2 (20.7) | | 118.6 (18.3) | | 121.4 (18.0) |
| Retinal nerve fiber layer temporal quadrant thickness | | 62.7 (12.6) | | 62.7 (11.9) | | 64.4 (11.6) |
| Vitreoretinal interface abnormalities | | 31 (8.8) | | 38 (7.0) | | 63 (3.9) |
| Retinal fluid or hemorrhage/structural alterations | | 55 (15.6) | | 39 (7.2) | | 67 (4.2) |
| *High-risk cohorts* | |  | |  | |  |
| Selection for high-risk breast/ovarian cancer cohort | | 30 (8.5) | | 60 (11.0) | | 263 (16.4) |
| Selection for high-risk lung cancer cohort | | 66 (18.8) | | 77 (14.2) | | 106 (6.6) |
| Selection for high-risk CVD cohort | | 215 (61.1) | | 73 (13.4) | | 155 (9.7) |
| *Laboratory values* | |  | |  | |  |
| Total cholesterol (mg/dL) | | 173.1 (44.6) | | 189.7 (38.9) | | 185.2 (38.1) |
| High-density lipoprotein (mg/dl) | | 49.9 (17.1) | | 59.3 (18.0) | | 60.4 (19.2) |
| Low-density lipoprotein (mg/dl) | | 87.4 (36.4) | | 103.6 (34.6) | | 100.7 (32.2) |
| Triglycerides (mg/dl) | | 187.1 (143.8) | | 138.3 (103.6) | | 122.3 (84.5) |
| Blood glucose (mg/dl) | | 148.7 (72.8) | | 94.1 (14.1) | | 87.6 (11.4) |
| Hemoglobin A1c (%) | | 7.3 (1.7) | | 5.9 (0.2) | | 5.2 (0.3) |
| Serum creatinine (mg/dl) | | 0.9 (0.7) | | 0.9 (0.3) | | 0.9 (0.2) |
| Glomerular filtration rate (mL/min/1.73 m2) based on Modification of Diet in Renal Disease Study equation | | 89.2 (26.5) | | 84.0 (19.1) | | 89.7 (19.0) |
| Alanine aminotransferase (U/L) | | 24.9 (19.3) | | 21.2 (13.7) | | 20.0 (12.8) |
| Aspartate aminotransferase (U/L) | | 22.2 (14.3) | | 21.8 (14.2) | | 21.1 (11.5) |
| Vitamin D (ng/ml) | | 27.0 (13.5) | | 30.0 (13.0) | | 30.0 (13.0) |
| C-reactive protein (mg/l) | | 5.6 (11.3) | | 3.1 (5.0) | | 2.2 (3.9) |
| Ratio between neutrophils (k/mcL) and lymphocytes (k/mcL) | | 2.4 (1.6) | | 2.3 (1.1) | | 2.3 (1.3) |
| Mean corpuscular hemoglobin concentration (% RBC) | | 32.6 (0.9) | | 32.6 (0.8) | | 32.9 (0.8) |
| Neutrophil segments (% WBC) | | 60.3 (9.9) | | 59.9 (9.4) | | 60.5 (9.0) |
| Total neutrophils (k/mcL) | | 4.5 (1.6) | | 4.0 (1.5) | | 3.8 (1.4) |
| Total lymphocytes (k/mcL) | | 2.2 (1.4) | | 1.9 (0.6) | | 1.8 (0.6) |
| Monocytes (% WBC) | | 6.6 (2.4) | | 6.8 (2.3) | | 6.6 (2.1) |
| Eosinophils (% WBC) | | 2.7 (2.1) | | 2.5 (2.2) | | 2.4 (2.2) |
| Basophils (% WBC) | | 0.8 (0.4) | | 0.7 (0.4) | | 0.7 (0.4) |
| Absolute monocytes (k/mcL) | | 0.5 (0.2) | | 0.4 (0.2) | | 0.4 (0.1) |
| Absolute eosinophils (k/mcL) | | 0.2 (0.2) | | 0.2 (0.2) | | 0.1 (0.1) |
| Absolute basophils (k/mcL) | | 0.1 (0.0) | | 0.0 (0.0) | | 0.0 (0.0) |
| Hemoglobin (g/dl) | | 14.0 (1.5) | | 14.1 (1.3) | | 14.3 (1.2) |
| Hematocrit (% RBC to whole blood volume) | | 42.9 (4.3) | | 43.2 (3.7) | | 43.4 (3.6) |
| Mean corpuscular volume (fL) | | 89.8 (6.3) | | 91.1 (6.5) | | 92.2 (5.1) |
| Mean corpuscular hemoglobin (pg) | | 29.3 (2.5) | | 29.7 (2.4) | | 30.3 (1.9) |
| Mean platelet volume (fL) | | 9.5 (1.0) | | 9.4 (1.0) | | 9.4 (1.0) |
| Platelet count (cumm) | | 257427.7 (71190.6) | | 246801.9 (61739.9) | | 242416.8 (60459.6) |
| Red blood cell count (millions/mcL) | | 4.8 (0.5) | | 4.8 (0.5) | | 4.7 (0.4) |
| White blood cell count (millions/mcL) | | 7.3 (2.4) | | 6.5 (1.9) | | 6.2 (1.7) |
| Total neutrophils (% WBC) | | 60.3 (9.9) | | 59.9 (9.4) | | 60.5 (9.0) |
| Total lymphocytes (% WBC) | | 29.6 (8.7) | | 30.1 (8.5) | | 29.7 (7.9) |
| Calcium (mg/dL) | | 9.5 (0.4) | | 9.5 (0.4) | | 9.5 (0.4) |
| Magnesium (MEQ/L) | | 1.6 (0.2) | | 1.7 (0.1) | | 1.7 (0.1) |
| Chloride (MEQ/L) | | 101.8 (2.8) | | 103.1 (2.5) | | 103.1 (2.2) |
| Potassium (MEQ/L) | | 4.3 (0.4) | | 4.3 (0.4) | | 4.3 (0.3) |
| Sodium (MEQ/L) | | 138.2 (2.3) | | 139.3 (2.1) | | 139.0 (2.0) |
| Protein in serum (g/dL) | | 7.0 (0.5) | | 7.0 (0.4) | | 7.1 (0.4) |
| Albumin (g/L) | | 4.3 (0.3) | | 4.3 (0.3) | | 4.4 (0.3) |
| Albumin urine detected (Y/N) | | 0.8 (0.4) | | 0.6 (0.5) | | 0.6 (0.5) |
| Uric acid (mg/dL) | | 5.5 (1.5) | | 5.3 (1.3) | | 5.0 (1.3) |
| Creatinine in urine (mg/dL) | | 109.1 (64.6) | | 106.8 (77.3) | | 101.6 (74.8) |
| Absolute reticulocytes (billions/L) | | 64.1 (26.0) | | 52.7 (22.3) | | 54.5 (22.5) |
| Reticulocytes (% RBC) | | 1.3 (0.5) | | 1.1 (0.5) | | 1.2 (0.5) |
| Thyroid-stimulating hormone (mIU/L) | | 1.9 (3.2) | | 1.8 (1.0) | | 1.8 (3.0) |
| Urine specific gravity | | 1.0 (0.0) | | 1.0 (0.0) | | 1.0 (0.0) |
| Urine reaction pH | | 5.9 (0.7) | | 6.2 (0.7) | | 6.4 (0.7) |
| *Physical performance measures* | |  | |  | |  |
| Age-adjusted 6-meter walk distance (meters) | | 511.0 (88.5) | | 532.0 (80.0) | | 583.4 (75.0) |
| Hand-grip strength (lbs) | | 74.5 (24.0) | | 76.3 (25.4) | | 78.6 (25.5) |
| Sit/rise score (range 0-10) | | 5.7 (2.4) | | 6.5 (2.4) | | 7.7 (2.1) |
| Single leg balance (seconds) | | 25.5 (22.4) | | 35.1 (22.3) | | 46.5 (19.8) |
| Ten meter walk speed (meters per second) | | 1.7 (0.4) | | 1.9 (0.5) | | 2.1 (0.5) |
| *Pulmonary function tests* | |  | |  | |  |
| Ratio of forced expiratory volume and forced vital capacity | | 0.8 (0.1) | | 0.7 (0.1) | | 0.8 (0.1) |
| Carbon monoxide diffusing capacity (DLCO) (mL/min/mmHg) | | 20.4 (7.6) | | 20.9 (5.9) | | 23.3 (7.3) |
| Forced expiratory volume 1 (FEV1) | | 2.7 (0.8) | | 2.8 (0.8) | | 3.2 (0.9) |
| Forced vital capacity (FVC) | | 3.5 (1.0) | | 3.7 (0.9) | | 4.2 (1.1) |
| DLCO% predicted | | 81.8 (22.3) | | 84.4 (26.4) | | 87.3 (26.5) |
| Verily Study Watch and surveys | |  | |  | |  |
| Average daily number of steps in the first 30 days in study (measured with Verily Study Watch) | | 6846.3 (3545.0) | | 8413.8 (3478.8) | | 8656.0 (3350.9) |
| Patient Health Questionnaire-9 total score (range 0, 27) | | 4.5 (4.4) | | 3.7 (4.2) | | 3.6 (4.1) |
| Alcohol Use Disorders Identification Test-Concise total score (range 0, 12) from first survey completed | | 1.5 (1.6) | | 2.0 (1.8) | | 2.3 (1.8) |
| Generalized Anxiety Disorder-7 total score (range 0, 21) | | 3.3 (4.4) | | 3.1 (4.1) | | 3.3 (4.1) |

Continuous variables are presented as the mean (SD), and categorical variables are presented as n (%). RBC, red blood cell; WBC, white blood cell.

**Additional Table S2.** PCA factors significantly associated overall with diabetes groups, with no differences between DM and noDM

|  |  | Overall association with diabetes groups | | Post-hoc pairwise comparisons,  p* | | |
| --- | --- | --- | --- | --- | --- | --- |
| Factor | **Primary variables** | **p** | **q†** | **DM vs. preDM** | **DM vs. noDM** | **PreDM vs. noDM** |
| Factor 25 | Left ventricular mass index (LVMI), left ventricular inner dimension end diastole (LVIDd), left ventricular inner dimension end systole (LVIDs), pulse (-) | 2x10^-7^ | 8x10^-7^ | 0.0004 | 1.0 | 2x10^-7^ |
| Factor 22 | Reticulocytes | 2x10^-6^ | 6x10^-6^ | 4x10^-6^ | 0.08 | 0.0001 |
| Factor 14 | Sodium, chloride | 4x10^-6^ | 1x10^-5^ | 2x10^-5^ | 0.2 | 9x10^-5^ |
| Factor 17 | Serum creatinine, chronic kidney disease (CKD), estimated glomerular filtration rate (eGFR) (-) | 6x10^-5^ | 0.0001 | 0.5 | 0.07 | 8x10^-5^ |
| Factor 13 | Ratio of FEV1 and FVC, oxygen saturation, chronic obstructive pulmonary disease (COPD) (-) | 0.0002 | 0.0005 | 0.02 | 0.9 | 0.0001 |
| Factor 29 | Systolic blood pressure, diastolic blood pressure | 0.0002 | 0.0005 | 0.2 | 0.4 | 0.0001 |
| Factor 15 | Coronary artery disease (CAD), history of myocardial infarction (MI), coronary artery calcium | 0.0009 | 0.002 | 0.2 | 0.6 | 6x10^-4^ |
| Factor 26 | Left ventricular cardiac index, left ventricular cardiac output, pulse | 0.0009 | 0.002 | 0.008 | 0.9 | 0.001 |
| Factor 23 | Platelets, mean platelet volume (-) | 0.002 | 0.003 | 0.6 | 0.2 | 0.002 |
| Factor 5 | Calcium, albumin, serum protein | 0.02 | 0.02 | 0.02 | 0.3 | 0.09 |
| Factor 27 | Monocytes | 0.03 | 0.04 | 0.2 | 0.9 | 0.02 |
| Factor 7 | Average retinal nerve fiber layer (RNFL) thickness, specific RNFL thicknesses: inferior quadrant, superior quadrant, nasal quadrant, total macular volume | 0.03 | 0.04 | 0.9 | 0.07 | 0.2 |

DM, diabetes; preDM, prediabetes; noDM, no diabetes.

Primary variables have absolute loadings >0.4 for the factor. Variables designated “(-)” are negatively loaded; all others are positively loaded.

*Adjusted for multiple comparisons using Tukey’s post-hoc test.

†False discovery rate (FDR)-adjusted p-values.

**Additional Table S3a.** Individual variables with high loadings in PCA factors associated with diabetes categories in the multivariable models

|  |  |  | Least-squares means (SE) | | | Overall association with diabetes groups, p | Post-hoc pairwise comparisons, p† | | |  |
| --- | --- | --- | --- | --- | --- | --- | --- | --- | --- | --- |
| Variable | **PCA factors** | **Category*** | **DM** | **PreDM** | **NoDM** |  | **DM**  **vs. preDM** | **DM**  **vs. noDM** | **PreDM vs. noDM** | |
| Hemoglobin A1c (%) | Factor 9 | Monotonic | 7.2 (0.04) | 5.9 (0.03) | 5.3 (0.02) | 2x10^-296^ | <10^-296^ | <10^-296^ | <10^-296^ | |
| Blood glucose (mg/dl) | Factor 9 | Monotonic | 146.5 (1.7) | 94.3 (1.4) | 88.6 (1.1) | 7x10^-166^ | <10^-296^ | <10^-296^ | 8x10^-4^ | |
| Selection for high-risk cardiovascular disease cohort | Factor 3,  Factor 9 | Misc. | 0.6 (0.02) | 0.2 (0.01) | 0.1 (0.01) | 1x10^-87^ | <10^-296^ | <10^-296^ | 0.1 | |
| Body mass index (kg/cm2) | Factor 1 | Monotonic | 33.3 (0.3) | 29.9 (0.3) | 27.8 (0.2) | 2x10^-45^ | <10^-296^ | <10^-296^ | <10^-296^ | |
| Mean corpuscular  hemoglobin (pg) | Factor 8 | Concordant | 29.2 (0.1) | 29.3 (0.1) | 30 (0.1) | 2x10^-13^ | 0.7 | 5x10^-9^ | 3x10^-10^ | |
| Mean corpuscular  volume (fL) | Factor 8 | Concordant | 89.3 (0.3) | 89.9 (0.3) | 91.4 (0.2) | 2x10^-10^ | 0.2 | 3x10^-8^ | 1x10^-6^ | |
| Mean corpuscular  hemoglobin concentration (% RBC) | Factor 8 | Concordant | 32.7 (0.05) | 32.6 (0.04) | 32.8 (0.03) | 4x10^-8^ | 0.4 | 0.006 | 4x10^-8^ | |
| Forced vital  capacity (FVC) | Factor 2 | Monotonic | 3.7 (0.04) | 3.8 (0.03) | 3.9 (0.02) | 1.x10^-7^ | 0.04 | 3x10^-7^ | 0.001 | |
| Selection for high-risk lung cancer cohort | Factor 3 | Concordant | 0.1 (0.02) | 0.1 (0.01) | 0 (0.01) | 2x10^-7^ | 0.4 | 5x10^-6^ | 1x10^-4^ | |
| Current smoker | Factor 3 | Concordant | 0.2 (0.02) | 0.2 (0.02) | 0.1 (0.01) | 6x10^-6^ | 0.7 | 0.01 | 9x10^-6^ | |
| Single leg balance  (sec) | Factor 1 | Misc. | 35.7 (1) | 40.5 (0.8) | 41 (0.6) | 8x10^-6^ | 2x10^-4^ | 6x10^-6^ | 0.8 | |
| Forced expiratory  volume 1 (FEV1) | Factor 2 | Concordant | 2.8 (0.03) | 2.9 (0.03) | 3 (0.02) | 2x10^-5^ | 0.5 | 2x10^-4^ | 0.002 | |
| Waist circumference (cm) | Factor 1 | Misc. | 94.5 (0.5) | 93 (0.4) | 92.3 (0.3) | 1x10^-4^ | 0.02 | 8x10^-5^ | 0.2 | |
| Left ventricular inner dimension end systole (LVIDs) (cm) | Factor 10 | Misc. | 2.7 (0.03) | 2.8 (0.02) | 2.8 (0.02) | 2x10^-4^ | 0.02 | 1x10^-4^ | 0.2 | |
| Age-adjusted 6-meter walk distance (meters) | Factor 1,  Factor 2 | Misc. | 554.5 (2.1) | 561.2 (1.7) | 564 (1.3) | 3x10^-4^ | 0.02 | 2x10^-4^ | 0.3 | |
| Sit/rise score  (range 0-10) | Factor 1 | Misc. | 6.7 (0.1) | 7.1 (0.1) | 7.2 (0.1) | 8x10^-4^ | 0.01 | 5.0x10^-4^ | 0.7 | |
| Hemoglobin (g/dl) | Factor 2 | Concordant | 14 (0.1) | 14.1 (0.1) | 14.2 (0) | 0.001 | 0.9 | 0.01 | 0.006 | |
| Carbon monoxide  diffusing capacity  (DLCO) (mL/min/mmHg) | Factor 2 | Misc. | 21.1 (0.3) | 21.6 (0.3) | 22.2 (0.2) | 0.005 | 0.4 | 0.007 | 0.1 | |
| Hand-grip strength (lbs) | Factor 2 | Misc. | 75.3 (1) | 78.7 (0.8) | 78.8 (0.6) | 0.006 | 0.02 | 0.005 | 1 | |
| Red blood cell count  (millions/mcL) | Factor 2,  Factor 8 | Concordant | 4.8 (0.02) | 4.8 (0.02) | 4.8 (0.01) | 0.008 | 1 | 0.05 | 0.02 | |
| Best available left  ventricular ejection fraction (%) | Factor 10 | Misc. | 59.3 (0.2) | 58.9 (0.2) | 58.6 (0.1) | 0.04 | 0.4 | 0.04 | 0.4 | |

DM, diabetes; preDM, prediabetes; noDM, no diabetes; RBC, red blood cell.

Models are adjusted for age, sex, race, body mass index, systolic blood pressure, and history of hypertension. Results are shown for individual variables with high loadings (|loadings|>0.4) in PCA factors of interest; variables that are not significantly associated with overall diabetes categories in the multivariable models are not shown.

*Monotonic factors/*a priori* variables showed statistically significant differences between all pairwise comparisons (noDM-preDM, noDM-DM, and preDM-DM), with mean values for participants with prediabetes intermediate to those of the other two groups. Concordant factors/*a priori* variables did not show statistically significant differences between preDM-DM, but did show differences between preDM-noDM. Miscellaneous factors showed a mix of patterns: statistically significant differences between preDM-DM, but not between preDM-noDM (discordant); preDM participants with mean values intermediate to, but not significantly different from, both DM and noDM; and preDM participants with mean values more extreme than, and significantly different from, both DM and noDM.

†Adjusted for multiple comparisons using Tukey’s post-hoc test.

**Additional Table S3b.** *A priori* individual variables associated with diabetes categories in the multivariable models

|  |  | Least-squares means (SE) | | | Overall association with diabetes categories, p | Post-hoc pairwise comparisons, p* | | |
| --- | --- | --- | --- | --- | --- | --- | --- | --- |
| Variable | **Category** | **DM** | **PreDM** | **NoDM** |  | **DM**  **vs. preDM** | **DM**  **vs. noDM** | **PreDM vs. noDM** |
| ASCVD risk score | Misc. | 0.13 (0.004) | 0.06 (0.003) | 0.07 (0.002) | 2x10^-37^ | 2x10^-11^ | 2x10^-11^ | 0.04 |
| Triglycerides (mg/dl) | Misc. | 174 (5.6) | 140 (4.6) | 132 (3.5) | 4x10^-10^ | 1x10^-6^ | 1x10^-10^ | 0.3 |
| Coronary artery calcium: <=100 vs. >100 | Misc. | 0.26 (0.02) | 0.18 (0.02) | 0.19 (0.01) | 0.001 | 0.001 | 0.003 | 0.8 |

ASCVD, atherosclerotic cardiovascular disease; DM, diabetes; preDM, prediabetes; noDM, no diabetes.

Models are adjusted for age, sex, race, body mass index, systolic blood pressure, and history of hypertension. Results are shown for individual variables with high loadings (|loadings|>0.4) in PCA factors of interest; variables that are not significantly associated with overall diabetes categories in the multivariable models are not shown.

*Monotonic factors/*a priori* variables showed statistically significant differences between all pairwise comparisons (noDM-preDM, noDM-DM, and preDM-DM), with mean values for participants with prediabetes intermediate to those of the other two groups. Concordant factors/*a priori* variables did not show statistically significant differences between preDM-DM, but did show differences between preDM-noDM. Miscellaneous factors showed a mix of patterns: statistically significant differences between preDM-DM, but not between preDM-noDM (discordant); preDM participants with mean values intermediate to, but not significantly different from, both DM and noDM; and preDM participants with mean values more extreme than, and significantly different from, both DM and noDM.

**Additional Table S4.** Individual variables with high loadings in PCA factors associated with reversion to no diabetes in adjusted models.

| **Variable** | **PCA factor** | **OR (95% CI)** | **p** |
| --- | --- | --- | --- |
| Total lymphocytes (k/mcL) | Factor 6 | 0.4 (0.3,0.6) | 2x10^-5^ |
| White blood cell count (millions/mcL) | Factor 6 | 0.8 (0.8,0.9) | 0.005 |
| Body mass index (kg/cm2) | Factor 1 | 0.95 (0.92,0.99)* | 0.006 |
| Waist circumference (cm) | Factor 1 | 0.97 (0.96,0.99) | 0.02 |
| Triglycerides (mg/dl) ** |  | 0.9 (0.9,1) | 0.03 |
| Sit / rise score (range 0-10) | Factor 1 | 1.1 (1,1.3) | 0.04 |

OR, odds ratio; CI, confidence interval.

OR > 1: higher levels of the variable are associated with higher odds of reversion from prediabetes to no diabetes; OR < 1: higher levels of the variable are associated with lower odds of reversion from prediabetes to no diabetes.

Models are adjusted for age, sex, race, body mass index (BMI), systolic blood pressure, and history of hypertension.

* Model is adjusted for age, sex, race, systolic blood pressure, and history of hypertension.

** Triglycerides were analyzed as an *a priori* variable of interest; the OR (95% CI) presented is for a 25 mg/dl increase
